# Low-Field Magnetic Resonance Imaging: A Full-Wave Simulation of Radiofrequency Birdcage Coils for Musculoskeletal Limb Imaging

**DOI:** 10.3390/diagnostics15060713

**Published:** 2025-03-12

**Authors:** Giulio Giovannetti, Francesca Frijia, Maria Filomena Santarelli, Vincenzo Positano

**Affiliations:** 1 CNR Institute of Clinical Physiology, 56124 Pisa, Italy; mariafilomena.santarelli@cnr.it; 2 Bioengineering Unit, Fondazione Toscana G. Monasterio, 56124 Pisa, Italy; f.frijia@ftgm.it (F.F.); positano@ftgm.it (V.P.)

**Keywords:** low-field magnetic resonance, radiofrequency coils, electromagnetic simulations

## Abstract

**Background:** Low-field Magnetic Resonance Imaging (MRI) (fields below 0.5 T) has received increasing attention since the images produced have been shown to be diagnostically equivalent to high-field MR images for specific applications, such as musculoskeletal studies. In recent years, low-field MRI has made great strides in clinical relevance due to advances in high-performance gradients, magnet technology, and the development of organ-specific radiofrequency (RF) coils, as well as advances in acquisition sequence design. For achieving optimized image homogeneity and signal-to-noise Ratio (SNR), the design and simulation of dedicated RF coils is a constraint both in clinical and in many research studies. **Methods:** This paper describes the application of a numerical full-wave method based on the finite-difference time-domain (FDTD) algorithm for the simulation and the design of birdcage coils for musculoskeletal low-field MRI. In particular, the magnetic field pattern in loaded and unloaded conditions was investigated. Moreover, the magnetic field homogeneity variations and the coil detuning after an RF shield insertion were evaluated. Finally, the coil inductance and the sample-induced resistance were estimated. **Results:** The accuracy of the results was verified by data acquired from two lowpass birdcage prototypes designed for musculoskeletal experiments on a 0.18 T open MR clinical scanner. **Conclusions:** This work describes the capability of numerical simulations to design RF coils for various scenarios, including the presence of electromagnetic shields and different load conditions.

## 1. Introduction

Magnetic Resonance Imaging (MRI) is a powerful and versatile imaging technique that generates images of the internal structures of the body. Conventional high-field MRI systems typically operate at field strengths ranging from 1.5 Tesla (T) to 7 T. However, low-field MRI (fields below 0.5 T) is achieving interest due to advancements in technology. Low-field MRI was initially used for its lower costs to manufacture, install, and maintain compared to high-field systems. Furthermore, lower magnetic field strengths reduce risks associated with MRI, such as “projectile effects” and radiofrequency-induced heating. Moreover, patients with contraindications for the use of high-field MRI, such as pacemakers or implants, may safely undergo low-field MR imaging [1,2,3].

In the musculoskeletal imaging field, several studies have indicated that higher field strength does not always lead to improved diagnostic performance. In particular, comparative studies between 1.5 T and 3 T have demonstrated only marginal advantages for 3 T scanners, with the production of images not necessarily translating into better diagnostic outcomes or therapeutic significance [4,5]. Low-field MRI plays a significant role in studying skeletal muscle physiology and pathology since the acquired image is less affected by susceptibility artifacts caused by tissues with varying magnetic properties. This makes low-field systems particularly advantageous for obtaining accurate muscle assessments in areas where high-field systems might encounter difficulties [6,7]. Recent studies have also shown that modern low-power MRI systems, thanks to the new hardware and software technologies available, have become very efficient and valid tools for diagnosis in the musculoskeletal system [8]. Moreover, such systems can also be used for real-time studies of skeletal muscle during exercise, which is particularly important for rehabilitation and understanding neuromuscular disorders.

In MRI, the signal-to-noise ratio (SNR) is directly proportional to the static magnetic field intensity. Hence, low-field MRI inherently has a lower SNR. To reach an acceptable SNR value, the image resolution should be reduced, or the scan time should be increased. On the other hand, low-field MRI is less affected by susceptibility artifacts. In fact, susceptibility artifacts decrease with T_2_* (effective transverse relaxation time), which is inherently longer in low-field MRI, meaning less signal dephasing and reduced susceptibility artifacts. Limitations of low-field MRI have been compensated by recent advances in hardware, such as improved gradient systems, high-performance radiofrequency (RF) coils, and noise reduction techniques. These developments allow low-field MRI to offer significant advantages in certain clinical and research settings [3,9,10]. In particular, RF coils are key components in low-field MRI systems. To optimize image quality, both transmit and receive coils have to be adapted to the specific anatomical target. Hence, the design of target-specific RF coils represents a key step in the development of low-field MRI systems. Numerical simulations play a crucial role in the design and optimization of RF coils. They are widely used to model and predict the performance of coil designs before physical prototyping, saving time and resources. In particular, numerical simulations can incorporate anatomical models to evaluate coil performance in realistic imaging scenarios, ensuring optimal signal transmission and reception for specific applications. The availability of software tools such as COMSOL Multiphysics, ANSYS HFSS, and XFdtd has allowed the development of advanced RF coils to accelerate, improving their efficiency, portability, and applicability in diverse settings. Recently, studies have been renewed to design more appropriate coils; these studies also include simulations in order to realize efficient coils even at low fields [11,12,13].

The birdcage coil is often employed for both transmission and reception and belongs to the volume coils group, with the ability to generate a highly homogeneous magnetic field in a large region surrounding the sample with a high SNR [14]. Such a coil is constituted by N “legs” connected at each end to two circular loops, called “end-rings”. Although the birdcage coils are resonators characterized by N/2 + 1 distinct resonant modes [15], the working resonant mode is usually the one that guarantees the best magnetic field homogeneity. A previous paper [16] contained a description of a low-frequency strip lowpass birdcage coil designed with a simulator employing Biot–Savart law magnetostatic numerical integration, which evaluated the magnetic field distribution in free space (unloaded coil) and estimated the effect of an RF shield on the coil tuning frequency. Such metallic shields can often be employed to prevent interactions with the bore scanner components. However, such a simulation approach is not able to estimate the actual magnetic field distribution inside the sample and does not permit performance of a proper SNR calculation necessary for taking into account the sample noise. A successive paper [17] proposed a low-frequency wire lowpass birdcage coil designed as the receiver coil in a vertical B_0_ MRI open scanner produced by Esaote Biomedica (E-Scan 0.18 T, Genova, Italy) and dedicated to musculoskeletal limb studies. This birdcage coil was successively modified to be used in the same vertical-bore MRI scanner in quadrature mode by combining the sinusoidal and the end-ring resonant modes [18]. Although MR experiments performed on this quadrature birdcage coil showed that both SNR and magnetic field homogeneity increased with respect to the linear birdcage, the decoupling level between the two channels was not verified.

In this paper, we describe the full-wave simulation of the two previously described strip and wire lowpass birdcage coils designed for limb image acquisition. Simulations were performed with the finite-difference time-domain (FDTD) method [19], to propose a tool for predicting coil image homogeneity and SNR. The proposed approach permits estimation of the magnetic field pattern for the coil in the loaded condition. The tool can simulate the sample properties in detail, including geometry and electromagnetic properties. Moreover, by exploiting the fact that the FDTD method provides simulation data also in the time domain, the sample-induced resistance can be estimated and compared to the coil losses, whose computation permits the estimate of coil SNR. Finally, the effect of the RF shield on the magnetic field pattern and the tuning frequency was verified, as well as the decoupling between the two channels of the quadrature coil. The accuracy of the full-wave simulation result was compared with measurements on two birdcage prototypes, previously tested at the workbench and with MR acquisitions using a 0.18 T MR scanner. In particular, in [17], we compared the designed wire birdcage coil with a state-of-the-art commercial solenoid employed in clinical MR acquisitions, and we emphasized that birdcage provided better field homogeneity along the transversal plane and longitudinal plane in MRI phantom acquisitions.

In this novel paper, we first demonstrated that knowledge of the coil, sample-induced resistances, coil inductance, and the magnetic field pattern in loaded conditions permitted a complete coil performance characterization. Secondly, we showed that numerical simulations allowed prediction of the generated magnetic field without the need for real experiments for a defined anatomical target (i.e., the calf region).

The manuscript is organized as follows: after the description of the full-wave simulator (Section 2.1) and a brief summarization of the coil loss estimation methods employed in our study (Section 2.2), the strip birdcage (Section 2.3) and wire birdcage (Section 2.4) are introduced and described. Successively, the strip coil (Section 3.1) and wire coil (Section 3.2) simulation results are summarized. Finally, a discussion (Section 4) and conclusions (Section 5) follow.

## 2. Materials and Methods

### 2.1. FDTD Simulation

The coil full-wave simulations were performed with the FDTD algorithm by using the commercially available software XFdtd 7.8 (Remcom, State College, PA, USA). The birdcage coils were designed using the geometry workspace of the XFdtd tool, and the simulations were performed by modeling them using a Perfect Electric Conductor (PEC). An adaptive non-uniform mesh, finer in the coil’s conductor proximity, was used to minimize the computational time and load while achieving a good degree of accuracy. Finally, Perfect Matched Layer (PML) boundary conditions were used for truncating outward waves and mimicking an infinite computational domain [20]. Curved conductor surfaces were accurately modeled with a tool employing geometric information to provide a computational domain subcellular discretization for increasing the simulation accuracy for a given grid resolution and, in turn, reducing the memory constraints and the overall simulation time for a given level of desired accuracy. All the simulations were performed with automatic convergence detection, which was set to −90 dB, ensuring complete energy decay so that the signal died down sufficiently.

Initially, tuning capacitors were inserted in the coil legs, realizing a resonant circuit fed by an ideal current port. A Gaussian derivative pulse excitation in the feed port induced a damped voltage oscillation on the capacitor by permitting the resonance frequency evaluation. For the magnetic field pattern estimation, we simulated a non-resonant coil with a current feed (1 A) and a 50 Ω resistor using a sinusoidal wave of frequency equal to the desired Larmor frequency (*f_0_* = 7.66 MHz for 0.18 T proton MRI) placed in the coil legs through a 4 mm opening made in the structure of the conductor with a 45° phase shift between two adjacent current sources. Using these simultaneous excitations of all sources mimics the pattern that produces a homogeneous magnetic field in the coil and performs the function of the capacitors with the great advantage of computational time reduction [21,22]. Although such an ideal sinusoidal coil current is extremely accurate for an unloaded birdcage coil, this current distribution can be employed even when the simulated coil is operating at low Larmor frequencies and is loaded by a phantom, since the interaction between the coil and the phantom induces no significant additional currents on the coil conductors [23].

### 2.2. Coil Resistance and Sample-Induced Resistance Evaluation

The birdcage coil resistance can be derived by using the classic formula $R_{\mathrm{coil}}=\rho \frac{L_{\mathrm{len}}}{S}$, where ρ is the conductor resistivity (1.68 × 10^−8^ Ω·m for copper), and $L_{\mathrm{len}}$ and *S* are the total conductor length and cross-sectional area, respectively. It is necessary to take into account that, for alternating current (AC) resistance estimation, the current is considered confined in a region near the surface whose thickness is given by the penetration depth *δ* [24]:

(1) $$\delta =\sqrt{\frac{\rho }{\pi f_{0}\mu_{0}}}$$

where *μ*_0_ = 4π × 10^−7^ Henry per meter (H/m) is the free space permeability and $f_{0}$ is the coil tuning frequency.

The coil resistance of a birdcage (*N* legs, *h* length, *r* radius) constituted by a wire conductor of radius (*a*) can then be calculated as [25]:

(2) $$R_{\mathrm{coil}}=\frac{\rho \left(N\cdot h+4\pi r\right)}{2\pi a\delta }$$

The sample-induced resistance can be estimated by preliminarily calculating the coil quality factor *Q* as the ratio between the energy stored at the *i*-th cycle and the energy lost at the *i*-th cycle. By perturbing the birdcage coil with a Gaussian pulse, a voltage oscillation damped by the losses was measured. Then, the *Q* value was calculated by remembering that the energy stored by a capacitor is proportional to the square of the voltage across it [26]:

(3) $$Q=2\pi \frac{V_{i}^{2}}{V_{i}^{2}-V_{i+1}^{2}}$$

where *V_i_* and *V_i_*_+1_ are, respectively, the voltage at the *i*-th and (*i* + 1)-th cycles. With the birdcage simulation performed with a PEC conductor, the energy is dissipated only by the sample, and the sample-induced resistance $R_{\mathrm{sample}}$ can be evaluated as:

(4) $$R_{\mathrm{sample}}=\frac{2\pi f_{0}L}{Q}$$

where *L* is the birdcage inductance.

The birdcage inductance was estimated by considering the “global” inductance of the coil [27], in accordance with the definition by the transmission line approach [28].

All simulated birdcage coils were designed by choosing their dimensions for musculoskeletal experiments on a 0.18 T open MR clinical scanner.

### 2.3. Strip Birdcage Coil

**Figure 1:**
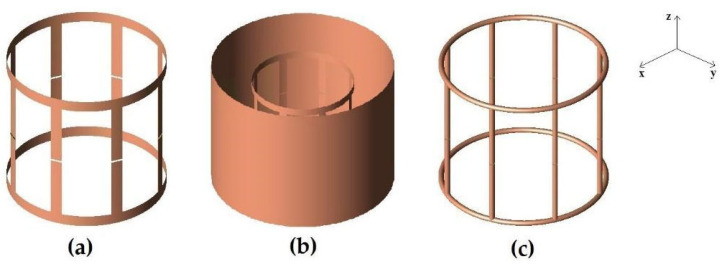
FDTD models of the simulated coils. (**a**) strip birdcage coil (8 legs, 11 cm length, 13.4 cm diameter) constituted by a 1 cm-wide strip. (**b**) strip birdcage coil as in (**a**) with a 16 cm diameter shield. (**c**) wire birdcage coil (8 legs, 11 cm length, 14 cm diameter) constituted by a 2.23 mm-radius wire.

**Figure 2:**
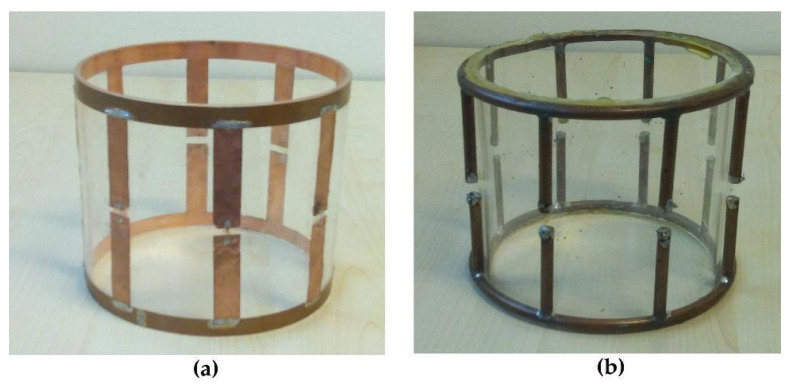
Picture of the home-built coils: (**a**) strip birdcage (8 legs, 11 cm length, 13.4 cm diameter, 1 cm-wide strip); (**b**) wire birdcage (8 legs, 11 cm length, 14 cm diameter, 2.23 mm-radius wire).

The first simulation was performed with a lowpass birdcage coil (8 legs, 11 cm length, 13.4 cm diameter) constituted by a 1 cm width strip (Figure 1a). The simulation was also carried out when the coil was shielded (Figure 1b). Two 18 cm-long PEC cylinders with a diameter of 16 and 24 cm, respectively, were considered in the simulation tool. A prototype of the coil (Figure 2a) was built by using a plexiglass cylinder and a 1 cm-wide and 35 µm-thick adhesive strip, then 2 nF capacities were introduced along the legs using high-quality non-magnetic ATC capacitors (American Technical Ceramics, Huntington Station, NY, USA). Successively, a coil resonant frequency workbench measurement was performed with a network analyzer HP3577 (Hewlett Packard, Palo Alto, CA, USA) by acquiring the S12 signal from a homemade dual-loop probe constituted by two pick-up circular loops partially overlapped to minimize the mutual inductive coupling (a condition obtained by separating the loop centers by 0.75 times their diameter [29]). These workbench tests were performed both for the unshielded and shielded strip birdcage. In this last configuration, two homemade cylinders constituted by conductor material were used with identical sizes to the simulated ones. Finally, we calculated the magnetic field profiles along the coil axis to compare the simulated magnetic field homogeneity of the birdcage coil with the two different shield radii.

### 2.4. Wire Birdcage Coil

#### 2.4.1. Linear Mode

The second simulation was performed with a lowpass birdcage coil (8 legs, 11 cm length, 14 cm diameter) constituted by a 2.23 mm-radius wire (Figure 1c). The magnetic field pattern evaluation was performed with the birdcage in loaded configuration, with a load consisting of a cylindrical homogeneous phantom (diameter 11 cm and length 20 cm) whose dielectric properties meet the ASTM (American Society for Testing and Material) criteria for MR phantom development (electrical conductivity = 0.6 S/m, permittivity = 80) [30].

A prototype of this wire birdcage coil (Figure 2b) was built and employed as a receiver coil for a vertical B_0_ MRI system (Esaote E-Scan 0.18 T, open MRI dedicated to musculoskeletal limb studies) [17]. For the MR acquisition, the birdcage was oriented with its axis perpendicular to the B_0_ field and loaded with a cylindrical homogeneous phantom of saline solution simulating the knee conductivity (diameter 11 cm and length 20 cm, 55 mM of NaCl and 5 mM of NiCl2). The following parameters were used for the T1-weighted spin-echo imaging sequence: TE = 18 ms, TR = 500 ms, slice thickness = 10 mm, FOV = 18 cm × 14 cm, number of signal averages = 1, pixel dimension = 0.7 mm. The magnetic field distribution of the wire birdcage coil was extracted from the acquired images. This comes from the fact that the phantom is highly homogeneous, and the coil magnetic field profiles can be directly estimated from the pixel values in the acquired images, since, as with volume coils, the expected B_1_ slowly varies across the imaging volume [31]. Successively, a second load was employed in the birdcage simulation, constituted by a human voxel model. This model was a volumetric model of an adult man (age 39, height 180 cm, weight 90 kg), based on the scans from the National Library of Medicine’s Visible Human Male project. The model consisted of 39 tissue types with assigned appropriate electric conductivity, relative permittivity, and mass density [32].

#### 2.4.2. Quadrature Mode

When birdcage coils are employed in horizontal-bore MRI systems (solenoidal magnets) [14], they can operate in quadrature mode to produce a circularly polarized magnetic field, reaching an SNR improvement of a maximum value of $\sqrt{2}\;$ [33]. However, when employed in vertical B_0_ MRI systems (open magnets), the performance of this quadrature birdcage coil fails since one of the B_1_ components is parallel to the B_0_ direction. Giovannetti et al. [18] designed and tested a lowpass birdcage coil able to generate a quadrature configuration by exploiting a radial B_1_ field (sinusoidal mode) and a longitudinal B_1_ field guaranteed by the resonant end-rings (end-ring mode), which behaved like a Helmholtz pair. To use this birdcage as a receiver coil in a vertical MRI system, a rectangular pick-up coil was coupled to a single mesh of the birdcage to detect the sinusoidal mode. The insertion of a capacitor on each end-ring generated two resonant loops, which produced a B_1_ field along the longitudinal axis of the coil. This magnetic field was perpendicular to both the sinusoidal mode and to the B_0_ field, providing a quadrature detection. The quadrature coil was built by modifying the previously described wire birdcage coil. The MR acquisitions, performed by using a 7 cm-diameter and 12 cm-long cylindrical homogeneous phantom constituted of 55 mM of NaCl and 5 mM of NiCl_2_ and the same spin-echo imaging sequence previously described, confirmed that quadrature coil provides better field homogeneity and a higher SNR with respect to the linear coil. In particular, SNR increased by 18% and 23% in the transversal and longitudinal planes, respectively, while field homogeneity along the z-axis showed an increase of 35% with respect to the same linear coil [18].

In the present work, full-wave simulation was performed to measure the decoupling between the two quadrature channels. In particular, a current feed (with a sinusoidal waveform of amplitude 1 A and frequency of 8 MHz) with a resistor (50 Ω) was inserted in one leg opening, while, in an opening in the end-ring located at 90°, a passive load (50 Ω) was inserted to estimate the S12 scattering parameters, which represents the response measured at the output port on the second coil channel as a result of the signal incident on the input port on the first coil channel [34]. The 50 Ω resistor for both the current feed and passive load was employed since, generally, coils must be matched to 50 Ω in order to optimize the energy transfer through all parts of the spectrometer.

## 3. Results

### 3.1. Strip Birdcage Coil

Table 1 shows the FDTD results of the strip birdcage simulated resonant frequencies, both for the unshielded and shielded configurations, compared with MoM numerical simulation performed by Numerical Electromagnetic Code (NEC2) solver and with Magnetostatic Simulation. For the unshielded birdcage coil, the difference between the FDTD and MoM methods was about 3.5%, while the difference between FDTD and magnetostatic simulation was 1.7%. For the shielded birdcage coils, the differences between FDTD and magnetostatic simulation were 1.5% and 1% for 16 cm and 24 cm diameter shields, respectively.

**Figure 3:**
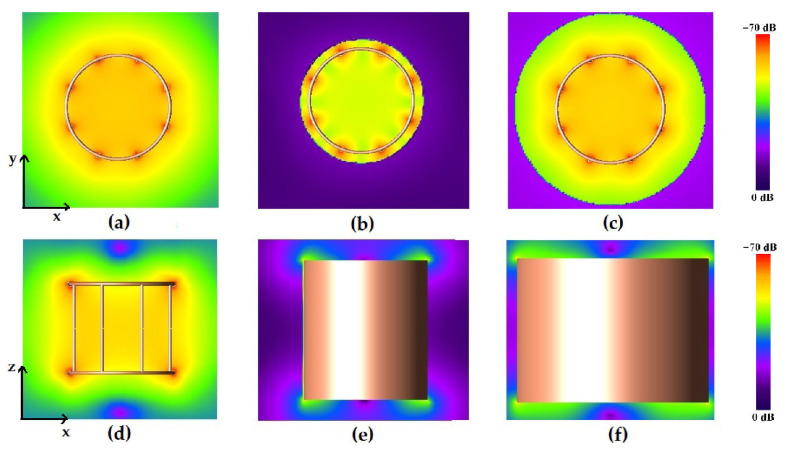
Magnetic field pattern for the strip birdcage coil: radial plane view for the unshielded (**a**), 16 cm-diameter shielded (**b**), and 24 cm-diameter shielded coils (**c**); longitudinal plane view for the unshielded (**d**), 16 cm-diameter shielded (**e**), and 24 cm-diameter shielded (**f**) coils.

Figure 3 shows the magnetic field pattern of the unshielded and shielded birdcage coil.

**Figure 4:**
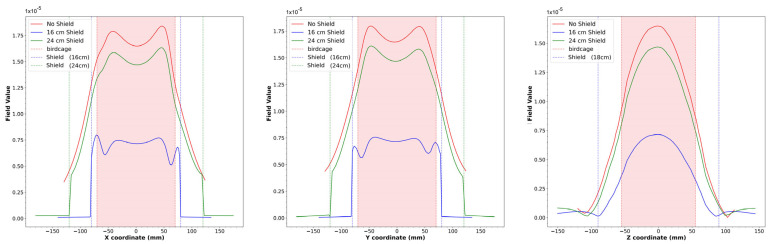
x, y, and z axis views for simulated magnetic field profiles for the unshielded strip birdcage coil, birdcage coil with 16 cm-diameter shield, and birdcage coil with 24 cm-diameter shield. The dotted lines represent the size of the birdcage (red) and the two shields (blue and green).

To compare the magnetic field homogeneity of the unshielded birdcage coil with the ones obtained with different shield radii, we extracted the profiles of the magnetic field along the coil axes; see Figure 4.

### 3.2. Wire Birdcage Coil

**Figure 5:**
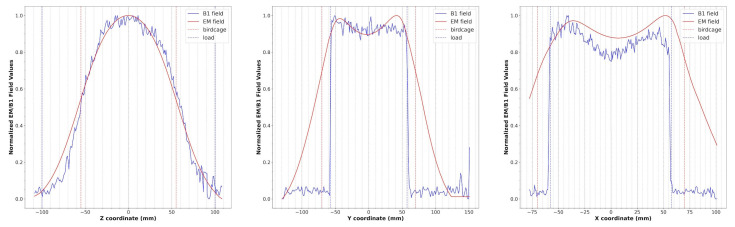
Simulated magnetic field profiles (red) and B_1_ field profiles (blue) extracted from phantom images along the z, y, and x axes for the wire birdcage coil. The dotted lines represent the size of the birdcage (red) and the load (blue).

The magnetic field modulus profiles along three straight lines passing through the birdcage center were estimated for the x, y, and z axes (Figure 5). In particular, the blue lines are obtained by extracting the profiles along each axis in the cylindrical phantom acquisition, while the red lines refer to the simulated magnetic field. Both profiles were normalized with respect to the maximum intensity of the B_1_ field and simulated magnetic field, respectively. The birdcage and phantom sizes are shown in the same figure as red and blue dotted lines, respectively.

Regarding the quadrature birdcage coil, the decoupling between the two quadrature channels resulted in S12 = −16.2 dB, lower than the commonly used threshold (−15 dB) that is considered an acceptable isolation level by MRI practitioners if preamplifier decoupling techniques [29] are applied.

**Figure 6:**
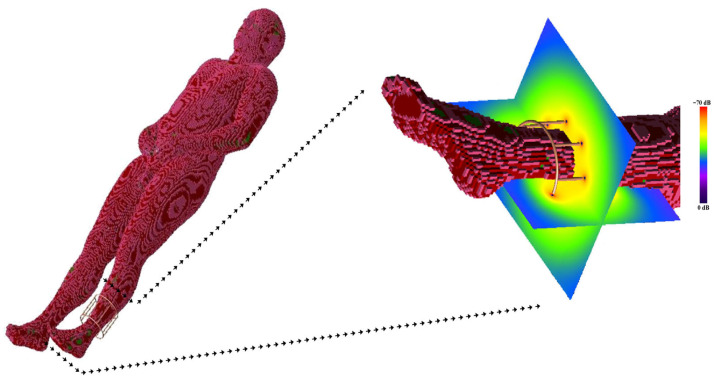
The birdcage coil in loaded condition with the human model and the magnetic field pattern associated with the coil in the calf region.

An electromagnetic simulation was performed to predict the RF field in the presence of an anatomical load suitable for an examination with the described coil geometry (i.e., the calf). Figure 6 shows the human model in the electromagnetic tool computational domain, together with the magnetic field pattern for the coil loaded with such a human model.

**Figure 7:**
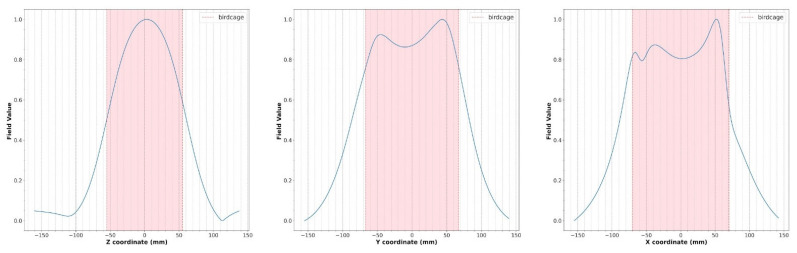
Simulated magnetic field profiles (blue) for the wire coil loaded with the human model (Figure 6). The size of the birdcage coil is indicated in light red.

Figure 7 shows the magnetic field profiles extracted from the planes depicted in Figure 6.

## 4. Discussion

Low-field MRI is a highly effective tool for studying skeletal muscle, offering non-invasive, detailed, and dynamic insights. Its low cost and greater accessibility compared to high-field systems make it an interesting setup for both research and clinical use.

For limb conditions, such as musculoskeletal injuries, arthritis, or tendon problems, the image quality provided by low-field MRI is sufficient in most cases for an accurate diagnosis. In fact, in the majority of clinical studies of the limbs, no significant difference in diagnostic accuracy between field strengths has been demonstrated [36,37,38]. Therefore, low-field MRI is well suited to the study of joint pathologies, such as for the evaluation of acute and chronic conditions of the elbow joint [39]; for the detection of wrist ligament injuries, differentiating occult fractures from bone bruises, or revealing avascular necrosis [40,41]; for the detection of extremities joint erosions [42]; and for knee meniscal and cruciate ligament tear diagnosis [43]. Moreover, there are cases in which low fields are preferable, such as imaging after prosthesis insertion: in the literature, many studies have been performed in postoperative evaluation, especially whenever complications are suspected. Prosthesis loosening, prosthesis infection, or other anomalies sometimes need to be evaluated, and these indications present a particular challenge for MRI due to metals causing signal artifacts [44], but these are much less pronounced at low-field strengths [45].

However, the low field strength limits the achievable SNR and the design of dedicated RF coils represents a fundamental step for effective musculoskeletal low-field MRI. In coil design, an accurate simulation process is necessary to avoid time-consuming trial-and-error approaches. In particular, the complete coil design should include the computation of the inductance, the magnetic field pattern in the loaded configuration, and the coil and sample losses. In this work, we proposed the application of an electromagnetic tool based on the FDTD method for the simulation, design, and performance prediction of low-frequency lowpass birdcage coils, both in unloaded and loaded configurations and with the presence of an RF shield.

Full-wave simulations showed that, as described in the literature [46], the insertion of the shield, by reducing the coil inductance values, increases the resonant frequency. As shown in Table 1, the coil resonant frequencies estimated with the FDTD method showed a deviation lower than 0.5 and 2.7% with respect to measured frequencies for the unshielded and shielded coil, respectively [16]. This deviation is compatible with the capacitor tolerances employed in the birdcage coil construction. For comparison, magnetostatic simulation [16] provided simulation data with a deviation lower than 2.2 and 4.2% with respect to measured frequencies for the unshielded and shielded coil, respectively, while MoM simulation [35] provided a deviation equal to 3.1% for the unshielded coil.

As can be seen in Figure 4, the presence of the shield slightly decreases the magnetic field homogeneity in the transverse plane, as yet verified with the method of images [47]. By comparing the plots of the simulated magnetic field for the loaded wire coil with those obtained from the experimental acquisition (Figure 5), it is possible to verify the great accuracy of the full-wave simulation.

The global inductance of the wire birdcage coil was derived by using the tuning frequency provided by the full-wave simulation (7.88 MHz) and by taking into account the calculated equivalent capacitance values [48], resulting in L = 120 nH. The coil quality factor, calculated with Equation (3), gave the result Q = 101. By inserting L and Q values into Equation (4), the sample-induced resistance gave the result 59 mΩ, while the coil resistance evaluated with Equation (2) was 91 mΩ at a frequency of 7.88 MHz.

These loss values showed that, at this low frequency, the SNR is strongly dependent on the coil losses, and an optimal coil design is a necessary constraint to minimize the coil noise with respect to the sample noise.

With respect to magnetostatic and MoM simulations, the FDTD method is very attractive because complex structures, such as parts of the human body, can be incorporated into the computational space, permitting us to obtain a biological sample–coil interaction model. To exploit this inherent advantage of FDTD simulation, we predict the RF field generated by a coil in the presence of an anatomical target (i.e., the calf) (Figure 6). As shown in Figure 7, the numerical simulator allowed us to predict the field variations induced by the anatomical load. We carried out comparison of the field shape in Figure 7 with the one obtained in the presence of a symmetric load (Figure 6). A field asymmetry induced by the asymmetric load can be noticed in the x and y planes. Field prediction could represent an important advantage in the management of MR experiments, especially in the research field, as scanner time is a costly resource that must be optimized. As the efficiency of RF coils is a key aspect in low-field MR imaging, if an image quality issue appears during a scan, it may derail the entire planned session, resulting in expensive “wasted” scan time. Predicting the RF coils’ performance prior to the session could allow us to solve this problem.

The study presents some limitations. We did not perform a sensitivity analysis of the simulation method. The errors in the experimental measurements of the resonant frequencies can be estimated to be about 2%, while the ones associated with the magnetostatic simulations can be assumed to be negligible since the theoretical resonant frequencies were estimated from inductance calculations, performed by implementing analytical equations with well-defined parameters. Finally, MoM and FDTD method errors depend on the mesh density and/or boundary condition setup, and, based on our experience, they can generally be made less than 0.1%.

## 5. Conclusions

High-performance, low-field MRI systems offer new clinical applications, particularly in musculoskeletal imaging. They can enhance image quality at critical air–tissue interfaces and in the presence of metallic implants. However, some intrinsic disadvantages of low-field MRI systems, such as the lower SNR, the reduced resolution, and the longer scan times compared to high-field MRI, should be compensated by enhanced system optimization. In particular, the design of target-specific RF coils can significantly improve performance. In this study, we demonstrate the ability of numerical simulations to predict RF coil performances in several scenarios, such as the presence of electromagnetic shields and different loads. Finally, the RF coil performances were predicted in the presence of a detailed anatomical target.

## Figures and Tables

**Table 1:** Unshielded and shielded strip birdcage resonant frequencies.

Experimental (f in MHz) [16]	FDTD Simulation (f in MHz)	MoM Simulation [35] (f in MHz)	Magnetostatic Simulation [16] (f in MHz)	Coil
8.08	8.12	7.83	8.26	Unshielded
10	10.27	n.a.	10.42	Shielded (d_sc_ = 16 cm)
8.41	8.45	n.a.	8.54	Shielded (d_sc_ = 24 cm)

## Data Availability

The raw data supporting the conclusions of this article will be made available by the authors on request.
